# Superiority of denosumab over bisphosphonates in preventing and treating glucocorticoid-induced osteoporosis: a systematic review and meta-analysis with GRADE quality assessment

**DOI:** 10.3389/fendo.2024.1407692

**Published:** 2024-12-18

**Authors:** Chiao-Ling Chen, Jian-Ying Wang

**Affiliations:** ^1^ Department of Pharmacy, Taipei City Hospital, Taipei, Taiwan; ^2^ Department of Pharmacy, New Taipei City Hospital, New Taipei City, Taiwan

**Keywords:** denosumab, bisphosphonates, glucocorticoid-induced osteoporosis, bone mineral density, meta-analysis

## Abstract

**Background:**

The increasing prevalence of glucocorticoid-induced osteoporosis (GIOP) due to long-term glucocorticoid therapy underscores the need for effective treatment options. Denosumab and bisphosphonates, both key in managing GIOP, require further comparative evaluation to determine their relative efficacy and safety profiles.

**Methods:**

We conducted a systematic review and meta-analysis, adhering to PRISMA guidelines. Our analysis included randomized controlled trials (RCTs) comparing denosumab with bisphosphonates in GIOP management. The outcomes were percent changes in bone mineral density (BMD) at various sites, bone turnovers markers (BTMs) and the incidence of adverse events.

**Results:**

Our study comprised five RCTs with 1,043 participants. The results showed a significant mean difference in BMD percentage change from baseline at LS of 2.87% (95% CI: 1.86 to 3.87, *p*<0.001) and at TH of 1.39% (95% CI: 0.15 to 2.64, *p*=0.03). Additionally, the safety profile of denosumab was found to be comparable to bisphosphonates, with no significant increase in the incidence of adverse events or serious adverse reactions.

**Conclusions:**

Denosumab proved more effective in enhancing BMD than bisphosphonates in GIOP, maintaining a comparable safety profile. However, the study’s limitations, including heterogeneity and the need for longer-term research, were noted.

## Introduction

In the U.S., statistics indicate that about 1.2% of the population uses glucocorticoids for prolonged durations, with a notably low frequency of antiosteoporotic medication use (1). Glucocorticoid-induced osteoporosis (GIOP) is a notable side effect of prolonged glucocorticoid therapy (2). This condition is characterized by a significant risk of bone loss, predominantly within the first few months of treatment (3). Studies have indicated that even low doses of prednisone or its equivalents, ranging from 2.5 to 7.5 mg daily, are associated with an increased risk of fractures (4). Therefore, GIOP requires aggressive management, especially in patients who are already at a higher risk for fractures, such as older individuals or those with a history of fragility fractures (3).

According to the 2022 guidelines of the American College of Rheumatology, it is recommended to assess fracture risk in patients aged 40 and over as soon as possible after starting a treatment with ≥2.5 mg/day of glucocorticoids (GC) for more than 3 months. This assessment should be done using the FRAX (Fracture Risk Assessment Tool) and by performing BMD testing using dual-energy x-ray absorptiometry (DXA) with vertebral fracture assessment (VFA) testing or spinal x-rays. The guidelines advise that all adult patients with medium, high, or very high fracture risk should be considered for osteoporosis therapy.

Bisphosphonates (BPs), widely used for managing osteoporosis and other bone diseases, work by inhibiting osteoclast activity, which are cells responsible for bone tissue breakdown (5). While they are a standard treatment for GIOP, oral BPs sometimes cause gastrointestinal side effects, such as esophageal irritation and ulceration, and have absorption issues (5). Although intravenous formulations can reduce gastrointestinal complications, intravenous BPs may present different side effects, including flu-like symptoms, fevers, myalgias, arthralgias, headaches, and also carry potential risks of atypical femoral fractures and osteonecrosis of the jaw (ONJ) (6). In comparison, teriparatide, another medication used for GIOP, has demonstrated better BMD improvements than BPs (7). However, its usage is generally restricted to no more than two years in a patient’s lifetime and necessitates frequent daily or weekly injections.

The challenge in treating GIOP arises from the effects of GCs on bone health. GCs increase the apoptosis of mature osteoblasts and osteocytes, leading to decreased bone formation and an impaired ability to respond to bone damage (8). They also extend the lifespan of osteoclasts by upregulating receptor activator of nuclear factor kappa-B ligand (RANKL) while suppressing osteoprotegerin (OPG) (8). Furthermore, GCs inhibit the Wnt signaling pathway—crucial for osteoblastogenesis—by increasing the expression of inhibitors like dickkopf-1 (Dkk-1) and sclerostin. This suppression results in reduced osteogenesis and poor bone regeneration (8).

Denosumab, a human monoclonal antibody, offers a more convenient option with biannual administration. It is effective in treating osteoporosis, bone loss from other diseases, bone metastases, and giant cell tumors of the bone (5). It functions as a RANKL inhibitor, preventing bone resorption by hindering the development of osteoclasts (5). In contrast, BPs primarily work by binding to bone to inhibit osteoclasts, acting more indirectly and relying on accumulation within the bone. In a murine model of GIOP, denosumab inhibited cortical bone loss without compromising biomechanical strength (9). This demonstrates its efficacy in preserving bone integrity under conditions that typically lead to significant bone loss (9).

Based on recent studies and systematic reviews, there is a growing body of evidence regarding the use of denosumab treating GIOP (10, 11). A 2022 systematic review and meta-analysis found that denosumab outperformed BPs in increasing lumbar spine BMD at 6 and 12 months, but both treatments showed similar improvements in total hip and femoral neck BMD (11). Additionally, while denosumab more effectively suppressed bone turnover markers like serum CTx (C-terminal telopeptide) and P1NP (procollagen type 1 amino-terminal propeptide), no significant differences in side effects, infections, or fractures were noted between the two treatments (11).

Based on the current evidence, we conducted an updated meta-analysis to explore the efficacy and safety of denosumab compared to BPs for GIOP. Furthermore, our analysis will include groups that have started using glucocorticoids for less than 3 months. A key focus of our study will also be on safety concerns, particularly the risk of infections. This is especially crucial since research indicates a higher risk of infection in postmenopausal women using denosumab, a concern that is magnified in populations with prolonged use of glucocorticoids. To ensure that our updated meta-analysis is reliable and clinically relevant, we employed the Grading of Recommendations Assessment, Development, and Evaluation (GRADE) framework (12). This approach enables a systematic evaluation of the quality of evidence, providing transparency in how conclusions are derived. The GRADE methodology was selected because it helps balance the benefits and risks of interventions, particularly in populations with complex health conditions such as GIOP. Additionally, it supports the prioritization of outcomes that matter most to patients and clinicians, such as fracture prevention and infection risk.

## Methods

### Systematic literature review

This systematic review and meta-analysis followed the Preferred Reporting Items for Systematic Reviews and Meta-Analyses (PRISMA) guidelines (13). It included randomized controlled trials (rcts) comparing the efficacy and safety of denosumab with BPs for prevention and treatment of GIOP. A comprehensive search was conducted in pubmed, Cochrane Library, and EMBASE, covering all publications until Jan 9, 2024. Search terms included “denosumab”, “bisphosphonates”, “Glucocorticoid-Induced Osteoporosis”, “Glucocorticoid”, “bone mineral density”, “Bone turnover”, “fracture” and related terms without language restrictions. Exclusion criteria were studies of less than 12 months duration or those not adhering to standard dosages. Two reviewers (C.L.C & J.Y.W) independently screened the results, resolving disagreements through discussion or a third party’s consultation. Funnel plots for publication bias assessment were used when the analysis included 10 or more studies.

### Quality assessment

Study quality was assessed using the Cochrane risk of bias (ROB) tool 2.0 (14), evaluating randomization process, deviations from intended interventions, missing outcome data, measurement of outcomes, and selection of reported results. Two independent reviewers (C.L.C & J.Y.W) conducted these assessments, with any discrepancies resolved through discussion and a third party’s consultation.

### Outcome, statistical methods and data synthesis

The primary outcomes for your study, as stated, will focus on the percentage changes in BMD at different sites - the lumbar spine (LS), femoral neck (FN), and total hip (TH) from baseline. Secondary outcomes encompassed risk of adverse events (AEs), including incidence of any AEs, serious AEs, infections. In addition, the occurrence of both vertebral and non-vertebral fractures was assessed.

Considering the expected variability in bisphosphonate usage, treatment duration, and patient demographics, a random-effects model was employed in the analysis. Heterogeneity was assessed using Cochran’s Q test and Higgins’s I^2^ statistic (15, 16). To assess the robustness of the effects in our analysis, we will employ a sensitivity analysis using the leave-one-out meta-analysis approach. Statistical analyses were performed using Review Manager Web (Revman Web) and STATA software (17, 18).

### Ratings of quality of evidence

The GRADE approach assessed the quality of evidence for each outcome, considering risk of bias, inconsistency, indirectness, imprecision, and other factors like publication bias and effect magnitude. We selected six main outcomes for evaluation, deemed essential for decision-making and focused on patient-important outcomes: percent change of BMD at LS, FN, TH, rates of any AEs, serious AEs, and any infection. The evidence was graded from very low to high and its importance categorized as critical, important, or nonimportant using GRADEpro/GDT software (12).

### Ethical statement

This systematic review did not involve direct human or animal subjects and therefore did not require ethical approval. All analyses were based on previously published data.

## Results

### Eligible studies and patient characteristics

**Figure 1:**
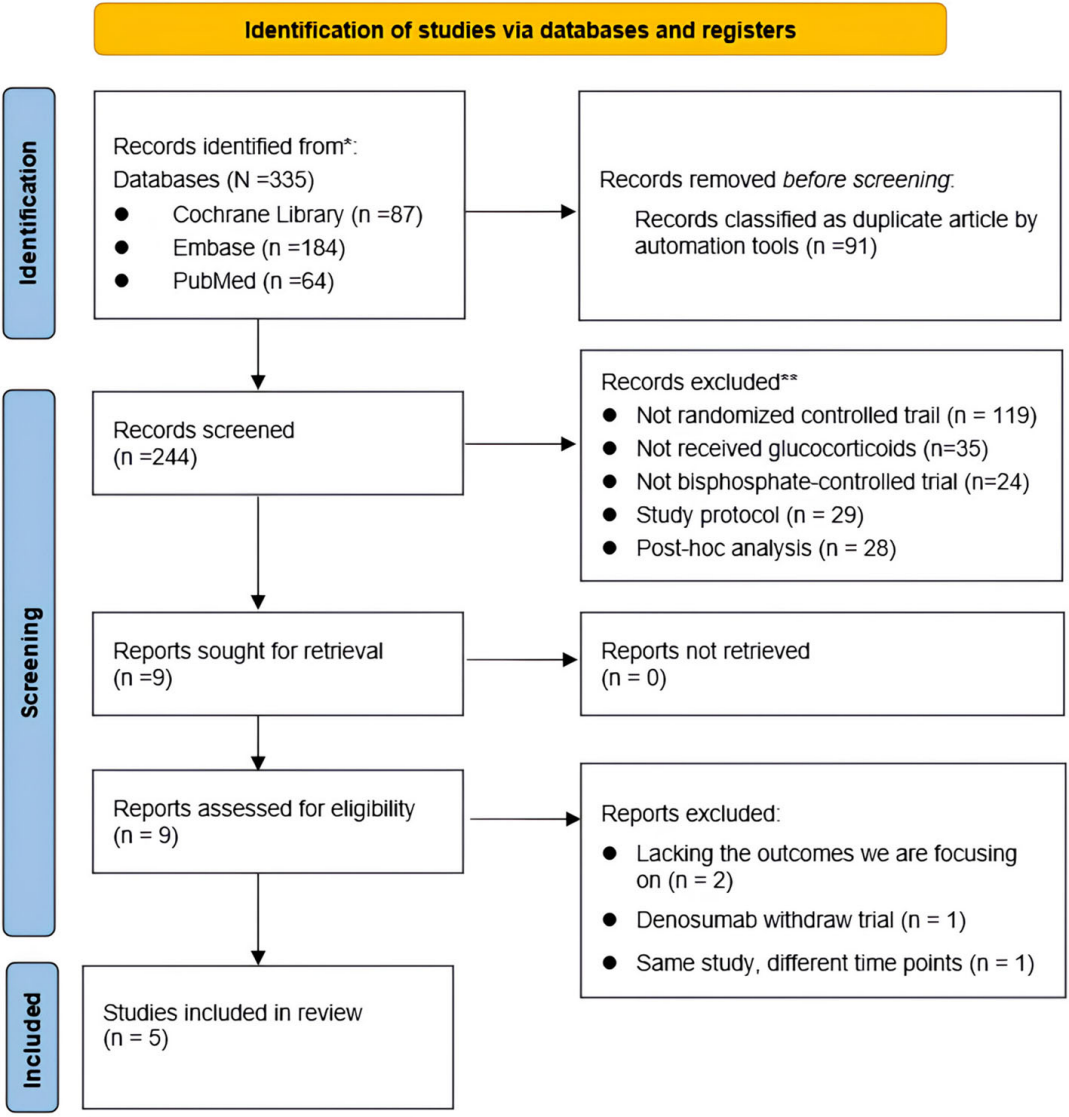
PRISMA flowchart for study selection process.

Our meta-analysis included 5 RCTs comprising 12 study arms with a total of 1,043 participants (19–23). A detailed flowchart of the screening process can be seen in **Figure 1**. The detailed characteristics of the included studies are presented in **Table 1**. Among these, two studies focused on populations previously treated with bisphosphonates, one was on treatment-naive individuals, and the rest did not specify or mixed populations. The age range of participants spanned from 48.0 to 68.5 years. In the treatment groups, denosumab was uniformly used at a dosage of 60 mg subcutaneously every 6 months. Among the comparator groups, two study arms involved varied bisphosphonates, two used alendronate, and two employed risedronate. The duration of the studies ranged from 12 to 24 months. The prednisolone equivalent dose ranged from 3 to 16.6 mg across the studies with treatment durations extending up to 111 months.

**Table 1:** Characteristics of the included trials.

Study	Eligibility Criteria	Treatment arms	Duration (months)	n	Age, years^a^	Female (%)	BMD, T-score^a^	Underlying disease	PSL daily dose, mg^a^	GC therapy duration (months)^a^
Iseri K, 2018 (23)	• Aged >20 years. • With glomerular disease on PSL. • Diagnosed with GIOP. • No prior bisphosphonate treatment.	DMB 60mg SC Q6M	12	14	66.5 (39.0-75.8)	42.9	LS: 0.895 (0.745-1.060), -1.3 (-2.5-0.3) FN: 0.672 ± 0.17, -1.3 ± 1.3	MCNS 4,Lupus nephritis 3, MN 2, ANCA-GN 3, FSGS 1, IgAN 1, HSPN 0	5.0 (2.4–8.5)	6.9 (2.2–19.0)
		ALN 35mg/week		14	65.5 (45.0-78.5)	42.9	LS: 0.875 (0.821–1.045), -1.2 (-1.9 – -0.4) FN: 0.627 ± 0.11; -1.7 ± 0.9	MCNS 5,Lupus nephritis 4, MN 3, ANCA-GN 0, FSGS 1, IgAN 0, HSPN 1	5.0 (2.5–9.3)	9.0 (1.8–19.1)
Mok CC, 2015 (20)	• Adult patients on long-term PSL (≥2.5 mg/day for ≥1 year) and oral BP (≥2 years)	DMB 60mg SC Q6M	12	21	54.9 ± 12.8	NA	LS: 0.830 ± 0.11,-2.27 ± 1.02 FN: 0.606 ± 0.08,-2.19 ± 0.70 TH: 0.731 ± 0.09, -1.73 ± 0.69	SLE 17, RA 4	4.60 ± 2.06	108.2 ± 56.0
		BP: ALN (79%), RIS (12%) and IBN (10%)		21	54.6 ± 13.4		LS: 0.810 ± 0.11, -2.47 ± 0.99 FN: 0.625 ± 0.09, -2.03 ± 0.79 TH: 0.748 ± 0.12, -1.61 ± 0.92	SLE 15, RA 6	4.12 ± 2.14	94.1 ± 75.6
Mok CC, 2021 (21)	• Adult patients on long-term PSL (≥2.5 mg/day for ≥1 year).	DMB 60mg SC Q6M	12	69	52.0 ± 12.3	99	LS: 0.858 ± 0.143, NR FN: 0.651 ± 0.111, NR TH: 0.774 ± 0.124, NR	SLE 81%, RA 9.4%, inflammatory myopathies 5% and systemic vasculitis 3.8%.	5.1 ± 2.9	111 ± 62
		ALN 70mg PO per week		70	48.0 ± 12.9	93	LS: 0.884 ± 0.170, NR FN: 0.677 ± 0.144, NR TH: 0.798 ± 0.148, NR		5.0 ± 2.4	104 ± 69
Saag KG, 2019 (GC-Continuing) (19)	• Aged ≥18 years, received ≥7.5 mg daily prednisone or equivalent for: ▪ <3 months (GC-initiating) or ▪ ≥3 months (GC-continuing) before screening. • All patients <50 years old with a history of osteoporotic fracture. • GC-continuing patients ≥50 years old with: ▪ T scores ≤ -2.0 or ▪ T scores ≤ -1.0 with fracture history.	DMB 60mg SC Q6M	24	253	61.5 ± 11.6	73	LS: NR, -1.92 ± 1.38 TH: NR, -1.66 ± 0.96	Rheumatological disorders 173, Respiratory disorders 46, IBD 3, Sarcoidosis 4, Neurological disorders 11, Dermatological disorders 9, Other 46	12.3 ± 8.09	0 to <3:13, ≥3: 239, missing data 1
		RIS 5 mg PO daily		252	61.3 ± 11.1	73	LS: NR, -1.96 ± 1.38 TH: NR, - 1.56 ± 0.96	Rheumatological disorders 184, Respiratory disorders 37, IBD 5, Sarcoidosis 5, Neurological disorders 15, Dermatological disorders 8, Other 37	11.1 ± 7.69	0 to <3:8, ≥3: 242, missing data 2
Saag KG, 2019 (GC-initialing) (19)		DMB 60mg SC Q6M		145	67.5 ± 10.1	64	LS: NR, -0.92 ± 1.86 TH: NR, -1.14 ± 1.00	Rheumatological disorders 129, Respiratory disorders 12, IBD 1, Neurological disorders 1, Dermatological disorders 6, Other 12	16.6 ± 13.01	0 to <3:133, ≥3: 10, missing data 2
		RIS 5 mg PO QD		145	64.4 ± 10.0	64	LS: NR, -1.06 ± 1.57 TH: NR, -0.98 ± 1.07	Rheumatological disorders 129, Respiratory disorders 11, Neurological disorders 2, Dermatological disorders 5, Other 11	15.6 ± 10.25	0 to <3:129, ≥3:16
Tamechika SY, 2023 (22)	• GC-treated SRD patients with: ▪ A pre-existing fragility fracture, or ▪ LS or FN BMD T-score ≤ -2.5, or ▪ T-score ≤ -1.5 without significant BMD increase in the past year despite oral BP therapy.	DMB 60mg SC Q6M	52 weeks	19	68.0 (58.0, 74.0)	68	LS: 0.737 (0.708, 0.835), −2.2 (−2.6, −1.5) FN: 0.523 (0.484, 0.557), −3.0 (−3.4, −2.6) TH: 0.638 (0.615, 0.689), −2.3 (−2.6, −1.9)	ANCA-associated vasculitis 3, Inflammatory myositis 8, AOSD 3, RA 2, SLE 1, GCA 0, Others 2	5.00 (3.00- 8.00)	>12 months
		BP (ALN 35 mg/week (35%], RIS 17.5 mg/week(15%], MIN 50 mg/4 weeks(50%])		20	67.0 (56.8, 72.0)	75	LS: 0.725 (0.659, 0.803), −2.5 (−2.9, −2.6) FN: 0.532 (0.481, 0.558), −2.9 (−2.6, −3.4) TH: 0.633 (0.564, 0.693), −2.5 (−3.1, −1.8)	ANCA-associated vasculitis 9, Inflammatory myositis 4, RA 1, SLE 2, GCA 2, Others 2	3.00 (2.50- 5.00)	>12 months

a Mean ± standard deviation or Median (interquartile range).

ALN, alendronate; ANCA-GN, anti-neutrophil cytoplasmic antibody glomerulonephritis; AOSD, adult-onset Still’s disease; BMD, bone mineral density; DMB, denosumab; FSGS, focal segmental glomerulosclerosis; FN, femoral neck; GC, glucocorticoids; GCA, giant cell arteritis; GIOP, glucocorticoid-induced osteoporosis; HSPN, henoch-schönlein purpura nephritis; IBN, ibandronate; IgAN, immunoglobulin A nephropathy; LS, lumbar spine; MCNS, minimal change nephrotic syndrome; MIN, minodronate; MN, membranous nephropathy; NR, not reported; PSL, prednisolone; Q6M, every six months; RA, rheumatoid arthritis; RIS, risedronate; SC, subcutaneous injection; SLE, systemic lupus erythematosus; TH, total hip.

### Quality assessment

The risk of bias assessment results for the studies can be found in **Supplementary Table S1**. After the evaluation, overall, four studies were classified as ‘some concern,’ mainly due to the lack of blinding for the medication. The remaining study, which used a double-blind, double-dummy design, was assessed as ‘high risk’ due to a higher dropout rate.

### The effects of denosumab versus BPs on BMD and bone turnover markers

**Figure 2:**
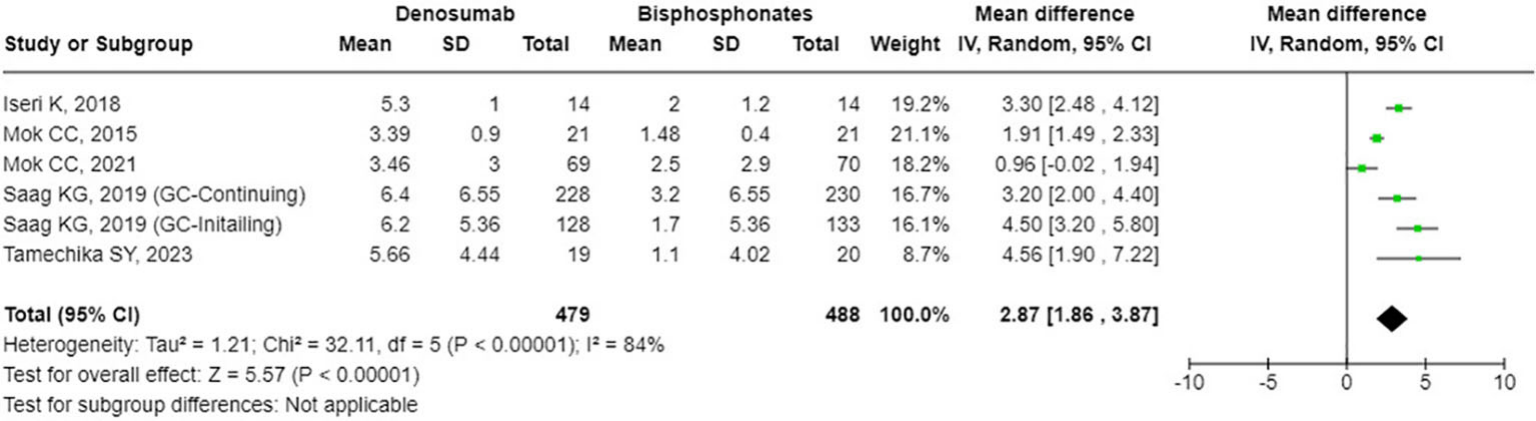
Forest plot of bone mineral density (BMD) percentage change from baseline at lumbar spine (LS) - denosumab vs. bisphosphonates.

**Figure 3:**
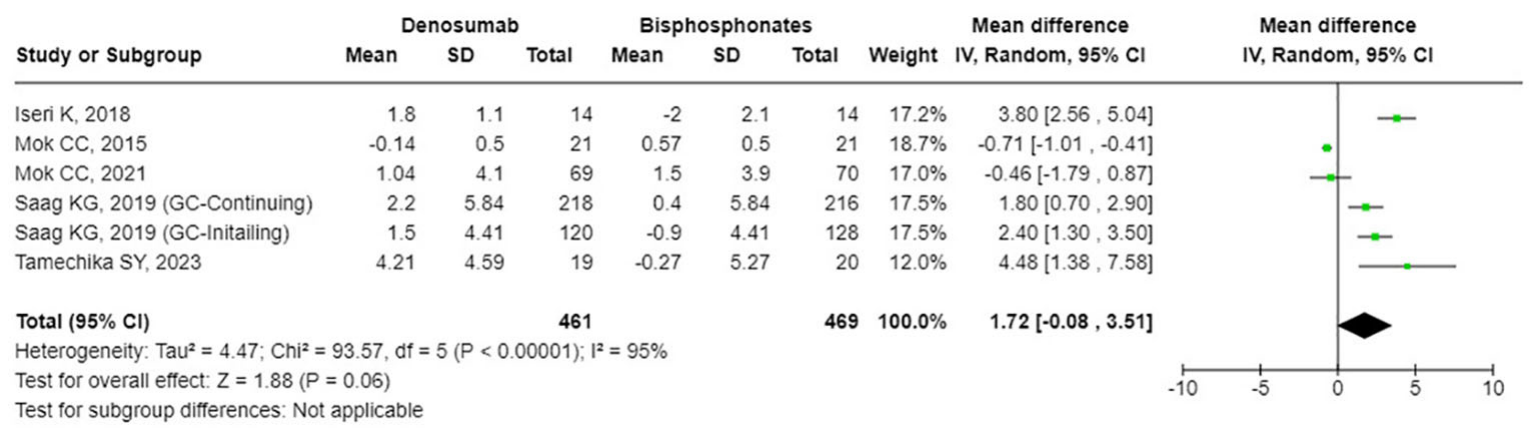
Forest plot of bone mineral density (BMD) percentage change from baseline at femoral neck (FN) - denosumab vs. bisphosphonates.

**Figure 4:**
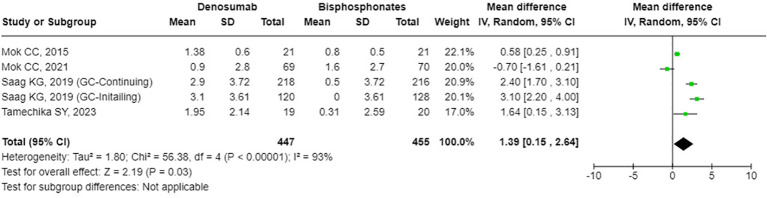
Forest plot of bone mineral density (BMD) percentage change from baseline at total hip (TH) - Denosumab vs. bisphosphonates.

The results from the random-effects model in our meta-analysis indicated significant differences in BMD changes when comparing denosumab with bisphosphonates. Specifically, denosumab demonstrated greater mean percentage changes in BMD from baseline at various sites: LS showed a mean difference of 2.87% (95% confidence interval [CI]: 1.86 to 3.87, p<0.001, I² = 84%, see **Figure 2**), the FN exhibited a mean difference of 1.72% (95% CI: -0.08 to 3.51, p=0.06, I² = 84%, see **Figure 3**), and the TH had a mean difference of 1.39% (95% CI: 0.15 to 2.64, p=0.06, I² = 93%, see **Figure 4**). Furthermore, the leave-one-out analysis indicated that the removal of any single study did not significantly affect the results for BMD at LS, FN, and TH.

Regarding bone turnover markers, we assessed four key indicators: P1NP, CTx, tartrate-resistant acid phosphatase 5b (TRACP-5b), and bone alkaline phosphatase (BAP). The findings showed that denosumab had a significantly higher percent change from baseline compared to BPs in P1NP, CTx, and TRACP-5b. Specifically, the mean differences were -26.93% (95% CI: -43.64 to -10.21, p=0.002, I² = 0%, see **Supplementary Figure S1**) for P1NP, -49.24% (95% CI: -75.97 to -22.51, p<0.001, I² = 0%, see **Supplementary Figure S2**) for CTx, and -26.37% (95% CI: -46.59 to -6.14, p=0.01, I² = 0%, see **Supplementary Figure S3**) for TRACP-5b. However, for BAP, there was no significant difference between the two treatments, with a mean difference of -11.96% (95% CI: -25.10 to 1.18, p=0.07, I² = 0%, see **Supplementary Figure S4**).

### Safety profile of denosumab versus bisphosphonates

**Figure 5:**
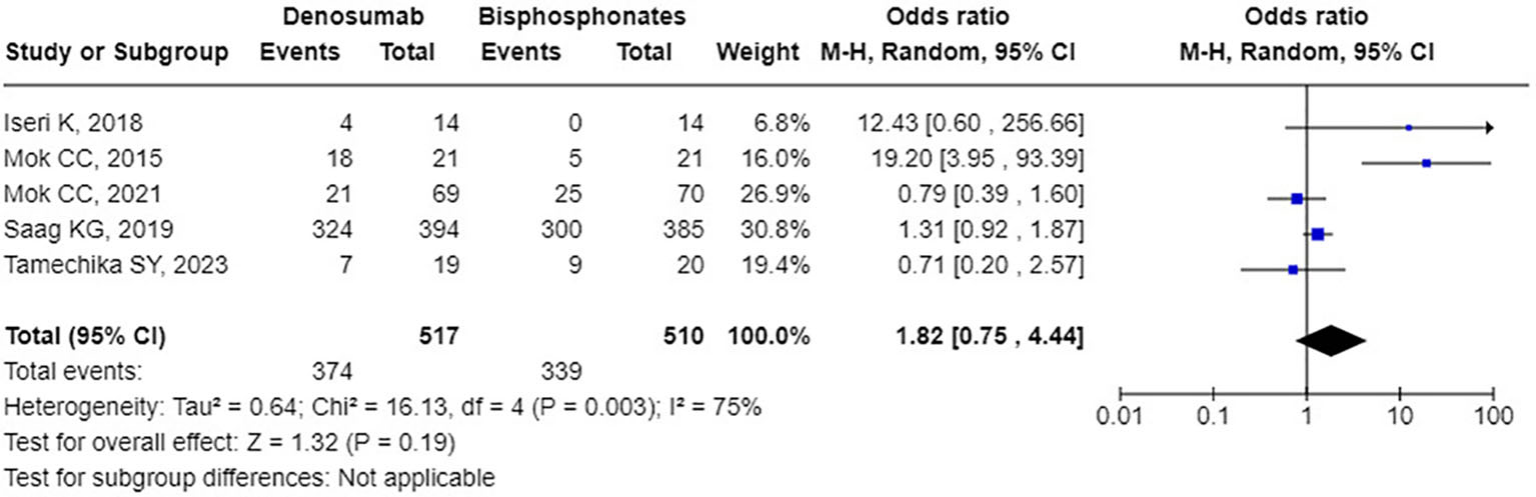
Forest plot of odds ratio for any adverse events - denosumab vs. bisphosphonates.

**Figure 6:**
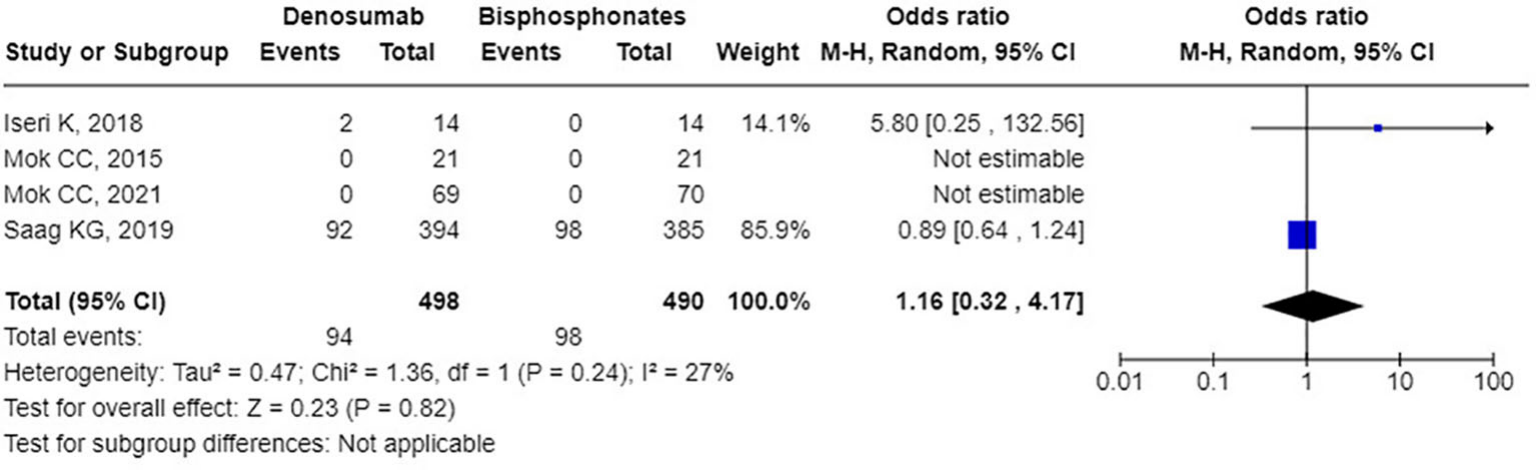
Forest plot of odds ratio for serious adverse reactions - denosumab vs. bisphosphonates.

Regarding safety, compared to bisphosphonates, denosumab did not significantly increase the odds ratio (OR) for any AEs, which was 1.82 [95% CI: 0.75 to 4.44], *p* = 0.19, I² = 75%, see **Figure 5**). Similarly, there was no significant increase in the odds ratio for serious AEs (OR: 1.16 [95% CI: 0.32 to 4.17], *p* = 0.82, I² = 27%, see **Figure 6**). Specifically, denosumab did not show a statistically significant increase in the odds for hypocalcemia (OR: 5.80 [95% CI: 0.25 to 132.56], p = 0.27, I² not applicable, see **Supplementary Figure S5**), any infection (OR: 1.39 [95% CI: 0.71 to 2.74], p = 0.34, I² = 29%, see **Supplementary Figure S6**), or serious infections (OR: 1.36 [95% CI: 0.63 to 2.93], p = 0.43, I² = 22%, see **Supplementary Figure S9**). Overall, common AEs related to denosumab in the trials included mild infections, hypocalcemia, back pain, joint pain, hypertension, and gastrointestinal symptoms. Serious adverse reactions included severe rashes, symptomatic hypercalcemia, and infections requiring hospitalization, although none resulted in fetal. Additionally, one case of atypical femoral fracture was reported, and no cases of osteonecrosis of the jaw were reported. Regarding fracture incidence, denosumab showed an odds ratio of 0.73 (95% CI: 0.34 to 1.56, p = 0.42, I² = 8%, see **Supplementary Figure S7**) for vertebral fractures; 1.39 (95% CI: 0.70 to 2.74, p = 0.34, I² not applicable, see **Supplementary Figure S8**) for non-vertebral fractures.

### Certainty of the evidence

In our GRADE assessment (detailed in **Supplementary Table S2**), specific limitations were identified that impacted the certainty of evidence for our outcomes. For the BMD Percentage Change at LS, FN, and TH, the risk of bias due to lack of blinding and dropout rates was a serious concern. High heterogeneity also influenced the certainty of evidence, alongside the small number of studies. When assessing the OR for any AEs, serious AEs, and any infection, similar issues were encountered. Consequently, the evidence was graded from very low to moderate, underscoring the need for careful interpretation in clinical decision-making for GIOP treatment.

## Discussion

Our meta-analysis offers critical insights into the efficacy and safety of denosumab in comparison to bisphosphonates, particularly in the context of pr GC therapy and long-term GC treatment. This analysis is especially pertinent given the diversity of patient groups, including those with prior bisphosphonate treatment, treatment-naive individuals. The marked improvement in BMD at the LS and TH with denosumab underscores its potential superiority over bisphosphonates, demonstrating notable effectiveness for both newly initiated and long-term GC users. This is of significant interest for patients initiating GC therapy, where early intervention is crucial for preventing GIOP. Although changes in BMD at the FN was less pronounced, they align with the trend favoring denosumab. Complementing our findings, the study by Geusens P et al. (24), although not included in our analysis, reveals similar trends in patients initiating GC therapy or on long-term GC therapy. Their study employed high-resolution peripheral quantitative computed tomography scans and found that denosumab was superior to risedronate in preventing failure load loss at the distal radius and tibia (24).

In terms of bone turnover markers, notably P1NP, CTx, and TRACP-5b, denosumab demonstrated a significant reduction compared to bisphosphonates. These findings are indicative of denosumab’s potent antiresorptive properties, essential in the early stages of GC therapy where bone turnover may be rapidly affected. In addition, changes BAP levels with denosumab versus BPs were not significant, contrasting with findings in postmenopausal women studies (25, 26). This warrants further investigation to understand these differing responses.

Regarding safety profiles, some studies have indicated that denosumab may increase the risk of infections (20, 27, 28). In populations undergoing long-term immunosuppressive therapy, the risk of infection is always a significant concern. Our study demonstrates that, in the GIOP population, denosumab has a similar safety profile to bisphosphonates, without increasing the risks of infection or fractures.

While our study confirms the efficacy and safety of denosumab, there are several limitations. Firstly, inherent methodological constraints, particularly non-uniform blinding protocols across included studies, necessitate a guarded interpretation of our outcomes. This lack of blinding introduces a potential for systematic bias, which is a common limitation in clinical trials, as noted in the literature. Secondly, the analysis revealed significant heterogeneity among the included studies, which may be attributed to variations in methodological designs. Differences in the types of BPs used, study durations, and patient demographics, such as comorbidities and the duration of GCs use, likely contributed to the observed inconsistencies. Although our leave-one-out sensitivity analysis underscored the stability of our results, the limited and selective pool of studies, particularly those of larger scale such as Saag KG et al., may skew the meta-analytical outcomes (19). Thirdly, the potential for publication bias remains a concern. The small number of included studies inherently limits the scope of our analysis and precludes comprehensive subgroup evaluations. This limitation constrains the extent to which our findings can be generalized across diverse clinical scenarios.

Fourthly, while our study included research with a maximum duration of 24 months, it’s important to consider the long-term implications of denosumab therapy. Research in postmenopausal women has demonstrated the efficacy and safety of denosumab over a 10-year period. This highlights a need for extended-duration studies to explore the long-term therapeutic outcomes and safety in patients with GIOP, including fracture risk. Moreover, the issue of discontinuation is crucial. As shown in the study by Saag KG et al., the focus was on rheumatoid arthritis patients receiving glucocorticoid treatment (29). The research found that upon discontinuation of denosumab, bone turnover markers and bone mineral density returned to baseline levels within a year (29). This emphasizes the need for ongoing bone health management in this specific patient group after stopping denosumab therapy. Lastly, although not directly addressed in this study, recent systematic reviews on cost-effectiveness analyses have indicated that most studies suggested denosumab is a cost-effective or superior option compared to oral bisphosphonates. This aspect is particularly important for future research in GIOP populations, especially regarding long-term use.

## Conclusions

In conclusion, our findings suggest denosumab may be a more effective option than BPs for patients on long-term glucocorticoid therapy, with a comparable safety profile. However, given the limitations observed, further studies, particularly larger and more diverse clinical trials, are necessary to fully understand denosumab’s role in this patient population. Future research should also focus on the long-term implications of denosumab use, including strategies for safe discontinuation and cost-effectiveness analysis.

## Data Availability

The original contributions presented in the study are included in the article/**Supplementary Material**. Further inquiries can be directed to the corresponding author.
