# Supplementary material for: Superiority of denosumab over bisphosphonates in preventing and treating glucocorticoid-induced osteoporosis: a systematic review and meta-analysis with GRADE quality assessment
Source: Front Endocrinol (Lausanne). 2024 Dec 18;15:1407692. doi: 10.3389/fendo.2024.1407692 (PMC11688227; doi:10.3389/fendo.2024.1407692)
Supplement: Supplementary file 1 [file DataSheet1.docx]

**Supplementary files**

**Table S1. Risk of bias summary**


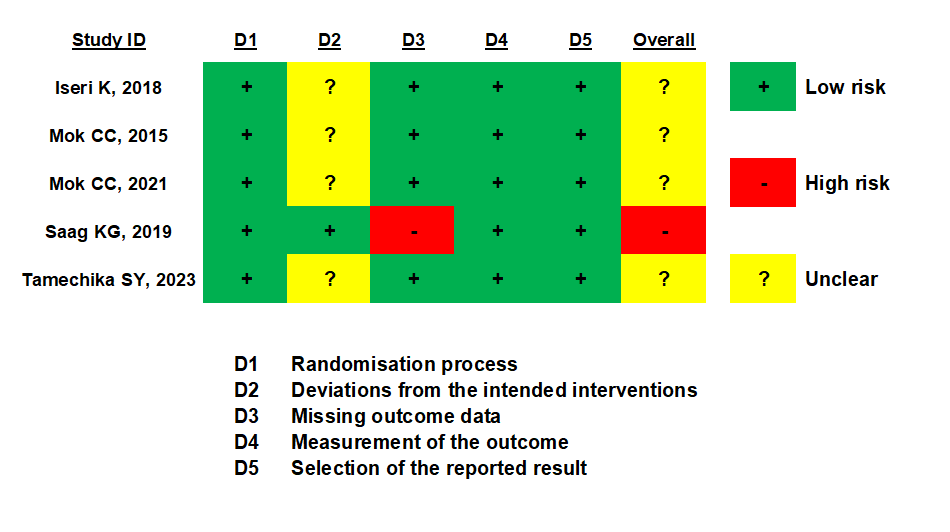


**Figure S1: Forest Plot of Mean Difference in Procollagen Type 1 N-Terminal Propeptide (P1NP) Levels - Denosumab vs. Bisphosphonates.**

**
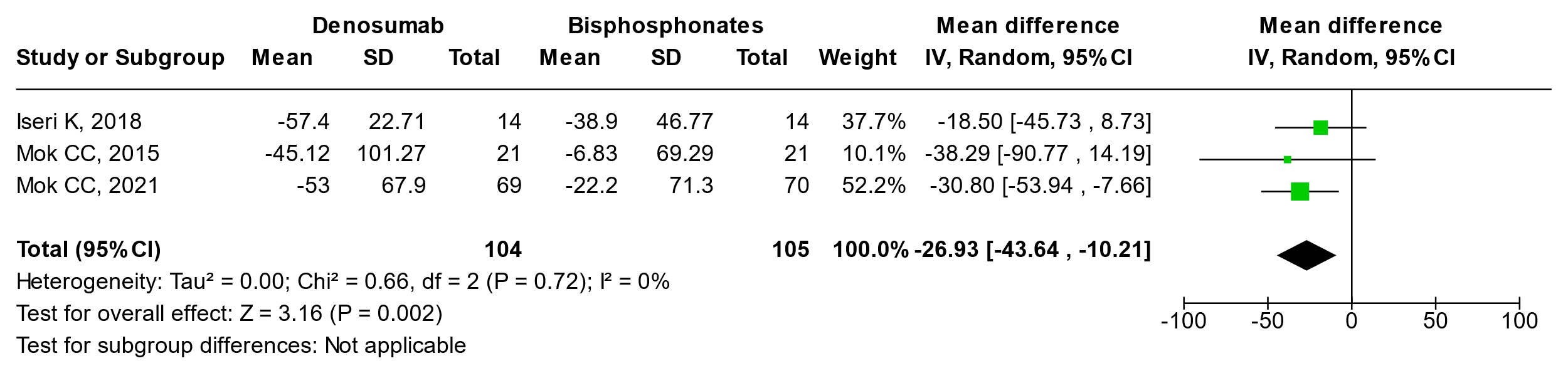
**

**Figure S2: Forest Plot of Mean Difference in C-Terminal Telopeptide of Type I Collagen (CTx) Levels - Denosumab vs. Bisphosphonates.**

**
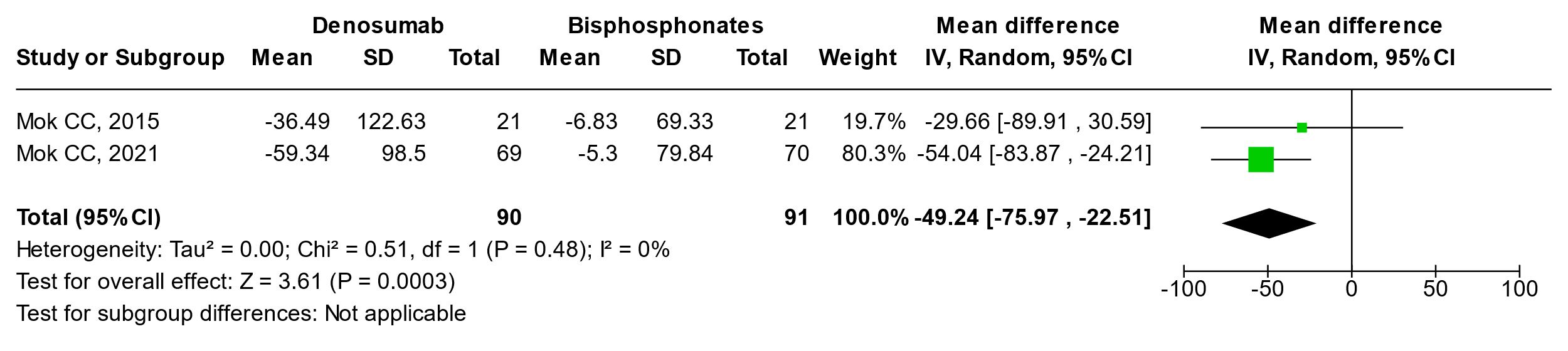
**

**Figure S3: Forest Plot of Mean Difference in Tartrate-Resistant Acid Phosphatase 5b (TRACP-5b) Levels - Denosumab vs. Bisphosphonates.**

**
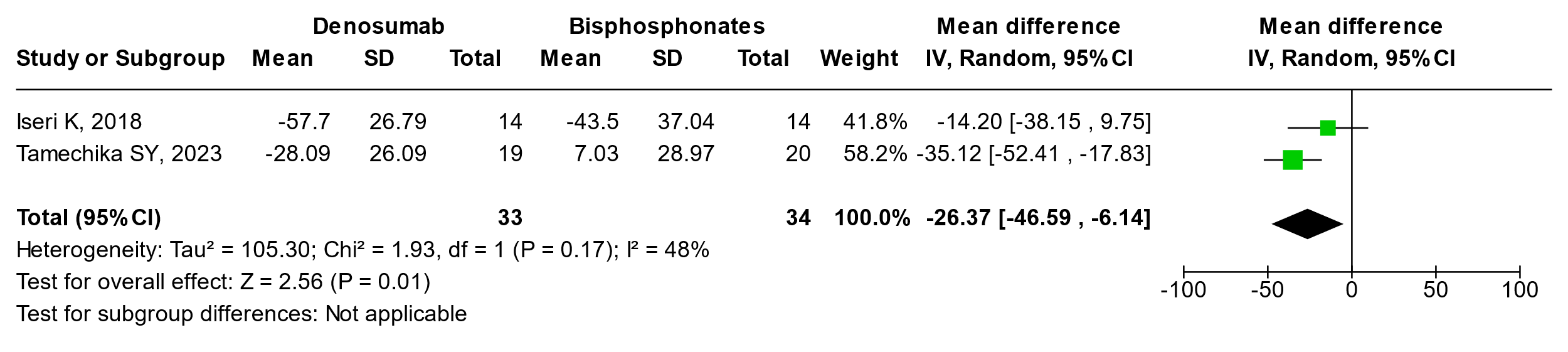
**

**Figure S4: Forest Plot of Mean Difference in Bone Alkaline Phosphatase (BAP) Levels - Denosumab vs. Bisphosphonates.**

**
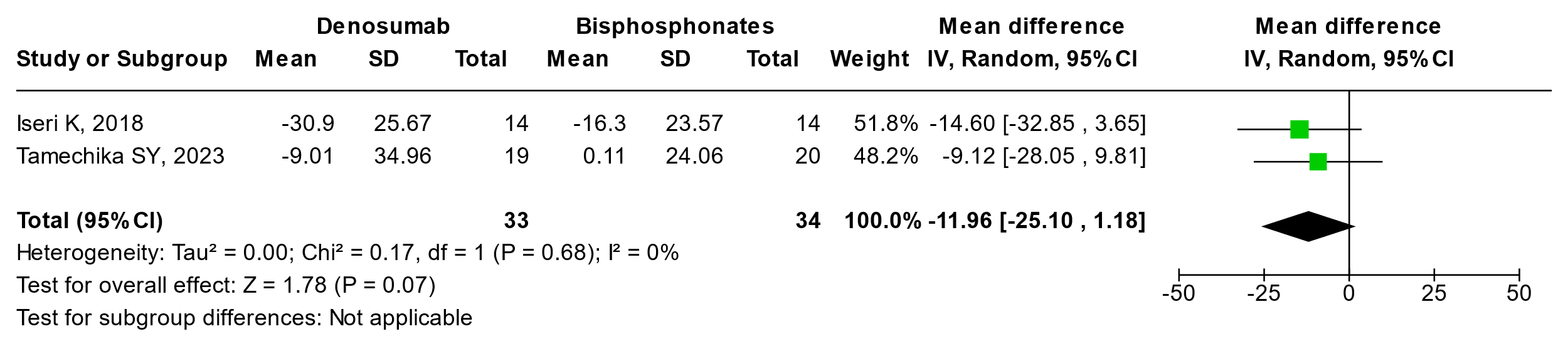
**

**Figure S5: Forest Plot of Odds Ratio for Vertebral Fractures - Denosumab vs. Bisphosphonates.
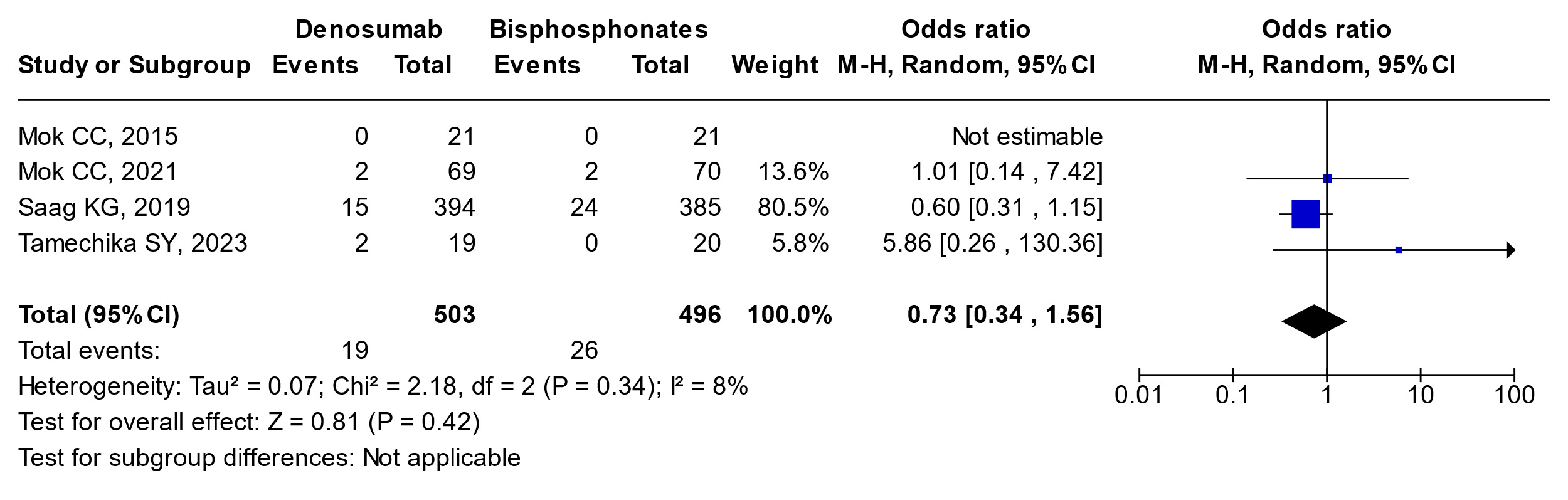
**

**Figure S6: Forest Plot of Odds Ratio for Non-Vertebral Fractures - Denosumab vs. Bisphosphonates.**

**
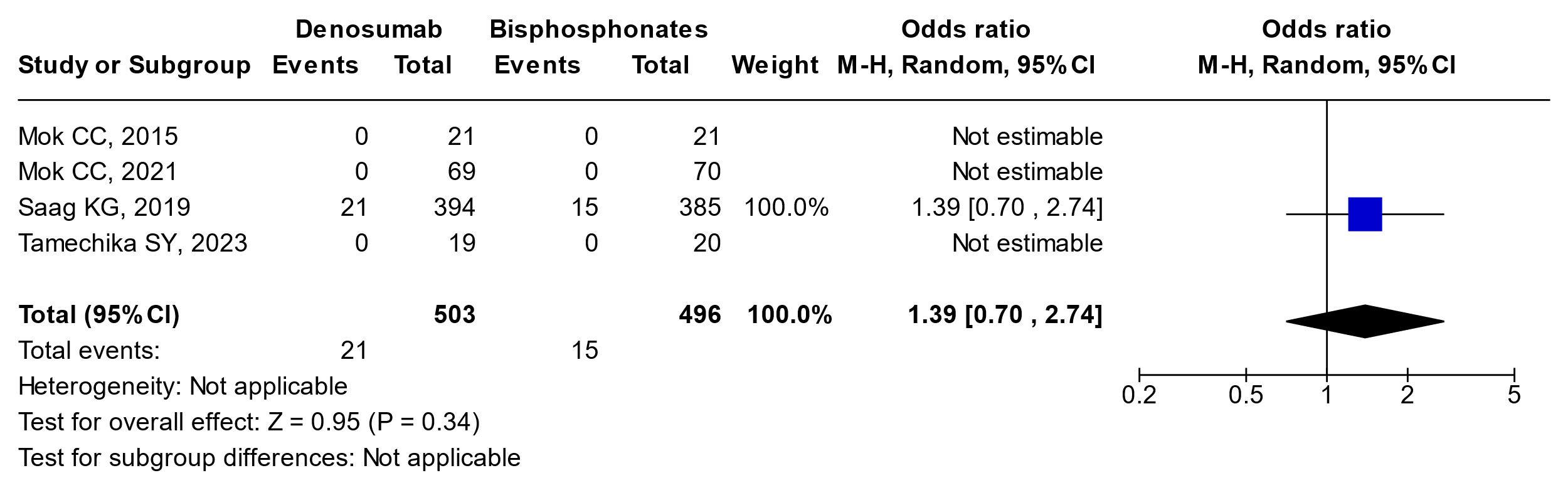
**

**Figure S7: Forest Plot of Odds Ratio for Hypocalcemia - Denosumab vs. Bisphosphonates.**

**
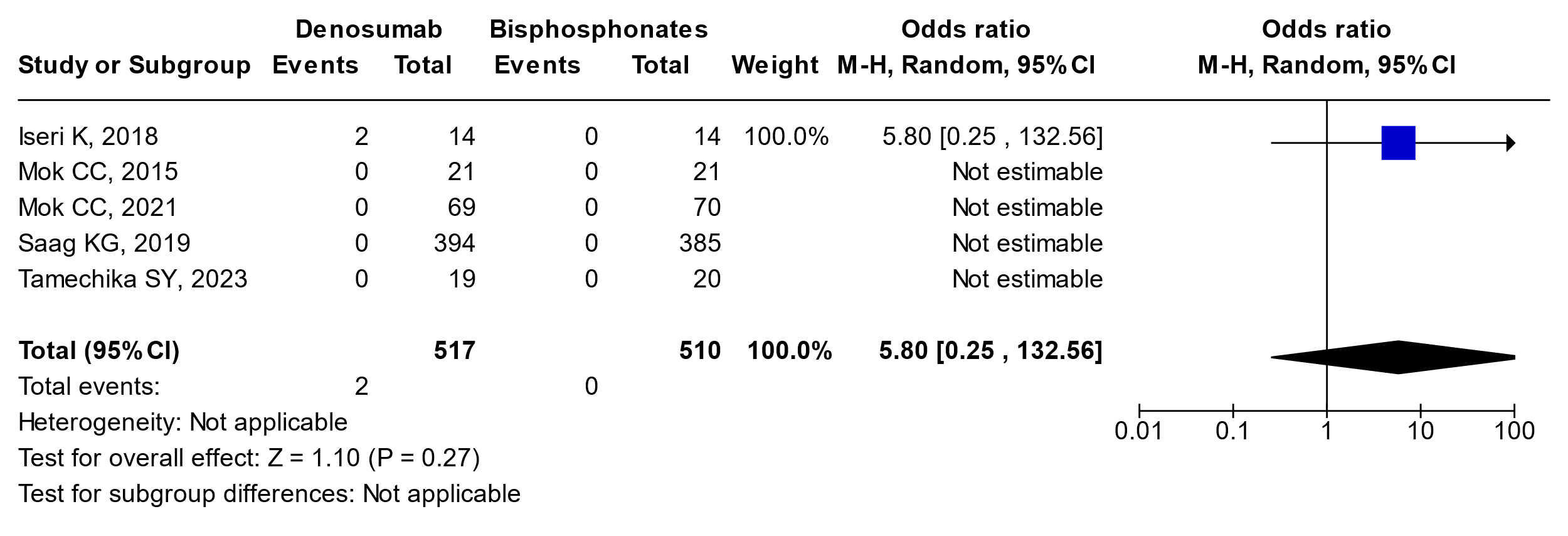
**

**Figure S8: Forest Plot of Odds Ratio for Any Infection - Denosumab vs. Bisphosphonates.**

**
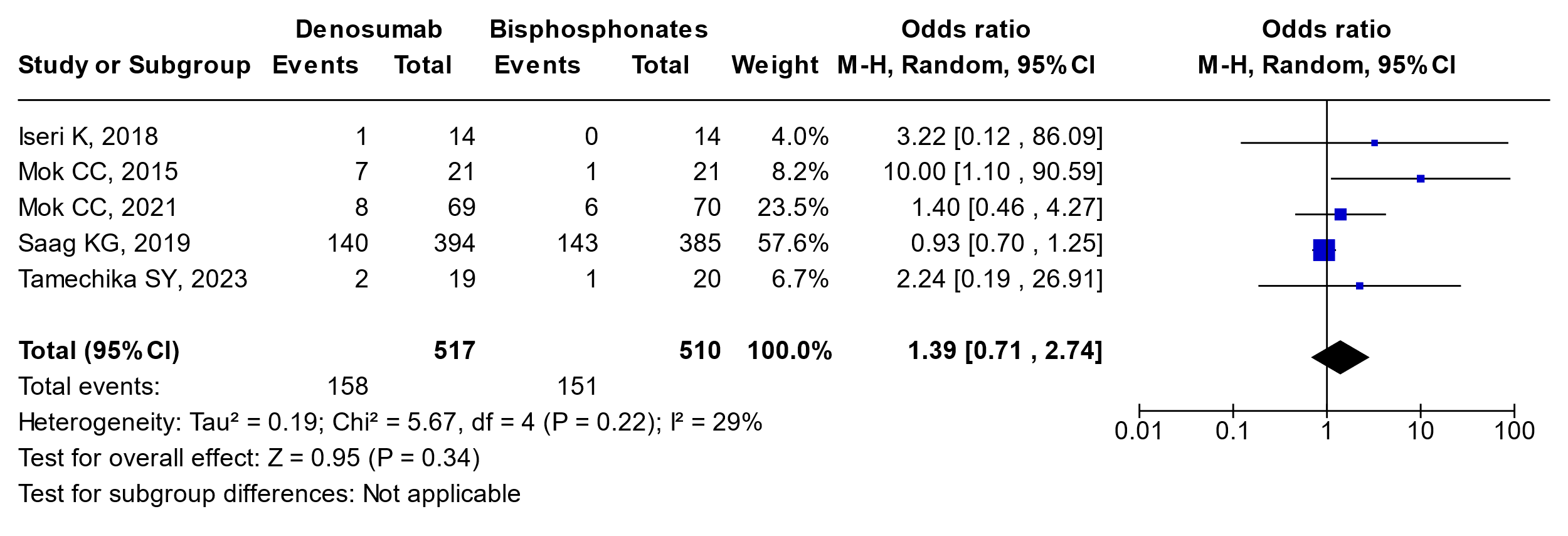
**

**Figure 9: Forest Plot of Odds Ratio for Serious Infections - Denosumab vs. Bisphosphonates.**

**
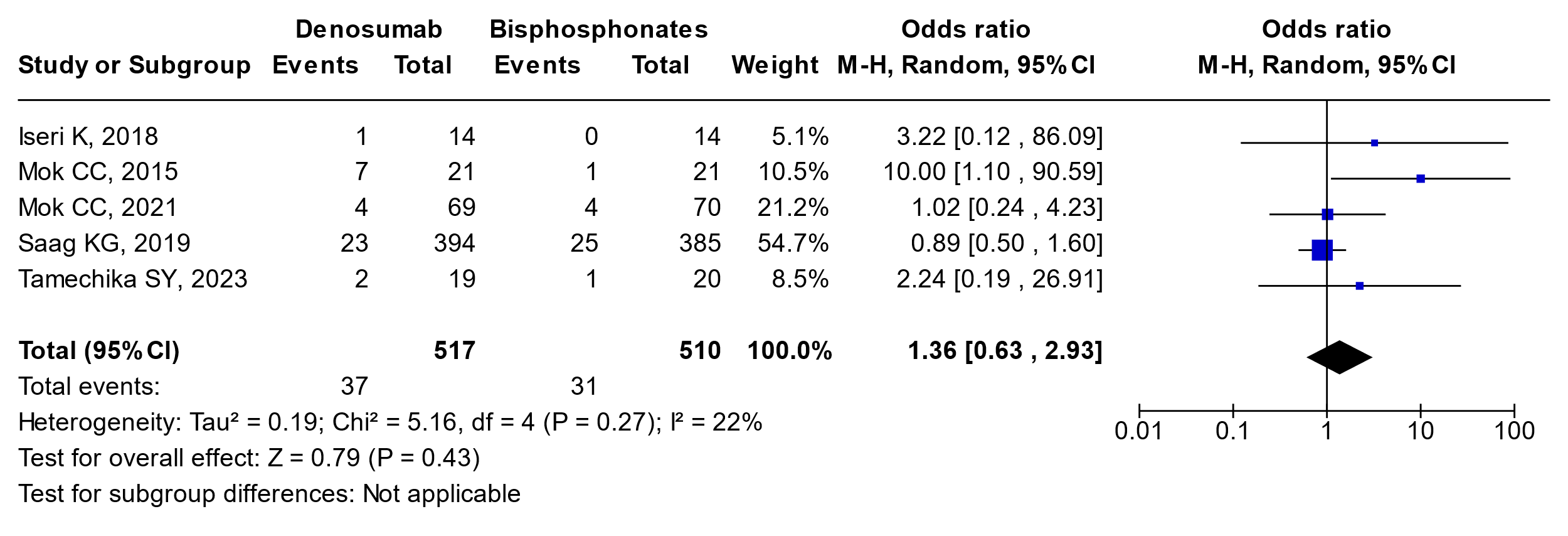
**

**Table S2. GRADE summary of findings table**

**Question:** Denosumab compared to Bisphosphates for Glucocorticoid-induced Osteoporosis (GIOP)

| **Certainty assessment** | | | | | | | **№ of patients** | | **Effect** | | **Certainty** | **Importance** |
| --- | --- | --- | --- | --- | --- | --- | --- | --- | --- | --- | --- | --- |
| **№ of studies** | **Study design** | **Risk of bias** | **Inconsistency** | **Indirectness** | **Imprecision** | **Other considerations** | **Denosumab** | **Bisphosphates** | **Relative (95% CI)** | **Absolute (95% CI)** |  |  |
| **Bone Mineral Density (BMD) Percentage Change from Baseline at Lumbar Spine (LS) - Denosumab vs. Bisphosphonates.** | | | | | | | | | | | | |
| 5 | randomised trials | serious^a^ | serious^b^ | not serious | not serious^c^ | publication bias strongly suspected very strong association^d^ | 479 | 488 | - | MD **2.87 higher** (1.86 higher to 3.87 higher) | ⨁⨁⨁◯ Moderate | CRITICAL |
| **Bone Mineral Density (BMD) Percentage Change from Baseline at Femoral Neck (FN) - Denosumab vs. Bisphosphonates.** | | | | | | | | | | | | |
| 5 | randomised trials | serious^a^ | serious^b^ | not serious | not serious^c^ | publication bias strongly suspected^d^ | 461 | 469 | - | MD **1.72 % higher** (0.08 lower to 3.51 higher) | ⨁◯◯◯ Very low | CRITICAL |
| **Bone Mineral Density (BMD) Percentage Change from Baseline at Total Hip (TH) - Denosumab vs. Bisphosphonates.** | | | | | | | | | | | | |
| 4 | randomised trials | serious^e^ | serious^b^ | not serious | not serious^c^ | publication bias strongly suspected strong association^d^ | 447 | 455 | - | MD **1.39 % higher** (0.15 higher to 2.64 higher) | ⨁⨁◯◯ Low | CRITICAL |
| **Odds Ratio for Any Adverse Events - Denosumab vs. Bisphosphonates.** | | | | | | | | | | | | |
| 5 | randomised trials | serious^a^ | serious^b^ | not serious | not serious | publication bias strongly suspected^d^ | 374/517 (72.3%) | 339/510 (66.5%) | **OR 1.82** (0.75 to 4.44) | **118 more per 1,000** (from 67 fewer to 233 more) | ⨁◯◯◯ Very low | CRITICAL |
| **Odds Ratio for Serious Adverse Reactions - Denosumab vs. Bisphosphonates.** | | | | | | | | | | | | |
| 4 | randomised trials | serious^e^ | not serious | not serious | not serious | publication bias strongly suspected^d^ | 94/498 (18.9%) | 98/490 (20.0%) | **OR 1.16** (0.32 to 4.17) | **25 more per 1,000** (from 126 fewer to 310 more) | ⨁⨁◯◯ Low | CRITICAL |
| **Odds Ratio for Any Infection - Denosumab vs. Bisphosphonates.** | | | | | | | | | | | | |
| 5 | randomised trials | serious^a^ | not serious | not serious | not serious | publication bias strongly suspected^d^ | 158/517 (30.6%) | 151/510 (29.6%) | **OR 1.39** (0.71 to 2.74) | **73 more per 1,000** (from 66 fewer to 239 more) | ⨁⨁◯◯ Low | CRITICAL |

**CI:** confidence interval; **MD:** mean difference; **OR:** odds ratio

#### Explanations

a. Four studies were classified as 'some concern,' mainly attributable to the lack of blinding, while one was assessed as 'high risk' due to a higher dropout rate.

b. High heterogeneity(I²>50%)

c. Total number of patients >400

d. Studies number <10

e. Three studies were classified as 'some concern,' mainly attributable to the lack of blinding, while one was assessed as 'high risk' due to a higher dropout rate.
